# Frequency shaping of tactile perception via transcutaneous interferential electrical stimulation: a simulation study

**DOI:** 10.3389/fnins.2026.1802332

**Published:** 2026-04-09

**Authors:** Hiroki Ohara, Shunsuke Yoshimoto, Yasuyoshi Asai, Tomohiro Ota

**Affiliations:** 1 Graduate School of Engineering, The University of Osaka, Suita, Japan; 2 Product Analysis Center, Panasonic Holdings Corporation, Kadoma, Japan

**Keywords:** axon cable equation, cable theory, computational simulation, electrotactile feedback, glabrous skin, Hodgkin–Huxley model, mechanoreceptor, transcutaneous electrical stimulation

## Abstract

Electrotactile feedback via transcutaneous interferential electrical stimulation generates temporally modulated stimulation fields and enables frequency-domain adjustment with carrier and beat frequencies. To systematically characterize these effects, we present a simulation framework that integrates tissue-scale electrical potential simulation with the finite element method and axon-scale dynamics using the axon cable equation, which incorporates cable theory and the Hodgkin–Huxley model, to predict axon activation and tactile perceptual metrics. We simulated a simplified glabrous skin model with three orthogonally oriented axons. Results show that the carrier frequency in the range of 1–4 kHz determines the upper bounds of the perceived field size, reaching up to 1.6 mm and exceeding the 1 mm electrode diameter, and perceived intensity, whereas the beat frequency in the range of 0–100 Hz adjusts these quantities within these bounds. Furthermore, axons oriented perpendicular to the skin surface exhibit lower activation thresholds than those oriented parallel. Unlike the conventional approaches of transcutaneous electrical stimulation, our results suggest that transcutaneous interferential electrical stimulation can shape the perceived field and perceived intensity without electrode reconfiguration or amplitude modulation. These findings clarify the distinct roles of carrier and beat frequency in tactile perception. This paper provides a theoretical foundation for frequency-domain adjustment of electrotactile interfaces and points toward compact, programmable systems. Quantitative validation through psychophysical experiments will further test and refine these predictions.

## 1 Introduction

The ability to render artificial tactile sensations is critical for enriching user experiences in human–computer interfaces, as virtual reality (VR) and augmented reality (AR) technologies become increasingly prevalent (Sundaram et al., 2019; Jung et al., 2022; Kang et al., 2025). For next-generation haptic systems, electrotactile feedback, which directly stimulates primary afferent fibers, has emerged as a promising approach

owing to its compact wearability, low power consumption, high temporal responsiveness and high spatial resolution (Lin et al., 2022; Yao et al., 2022; Huang et al., 2023; Xiong et al., 2025).

Among these techniques, transcutaneous electrical stimulation (TES) has been widely adopted because it is noninvasive. TES typically uses uniphasic or biphasic pulses at repetition rates up to approximately 200 Hz to recruit Aβ fibers, notably those linked to Meissner's corpuscles and Merkel complexes (Kourtesis et al., 2022; Ray et al., 2024). However, TES has key limitations in terms of sophisticated haptic rendering. The shaping of the perceived field (PF) or perceived intensity (PI) often requires electrode resizing (Kuhn et al., 2010) or amplitude calibration (Kajimoto et al., 1999), which either increases system complexity or the risk of nociceptive activation before sufficient tactile sensation is achieved (Ray et al., 2024).

**Figure 1:**
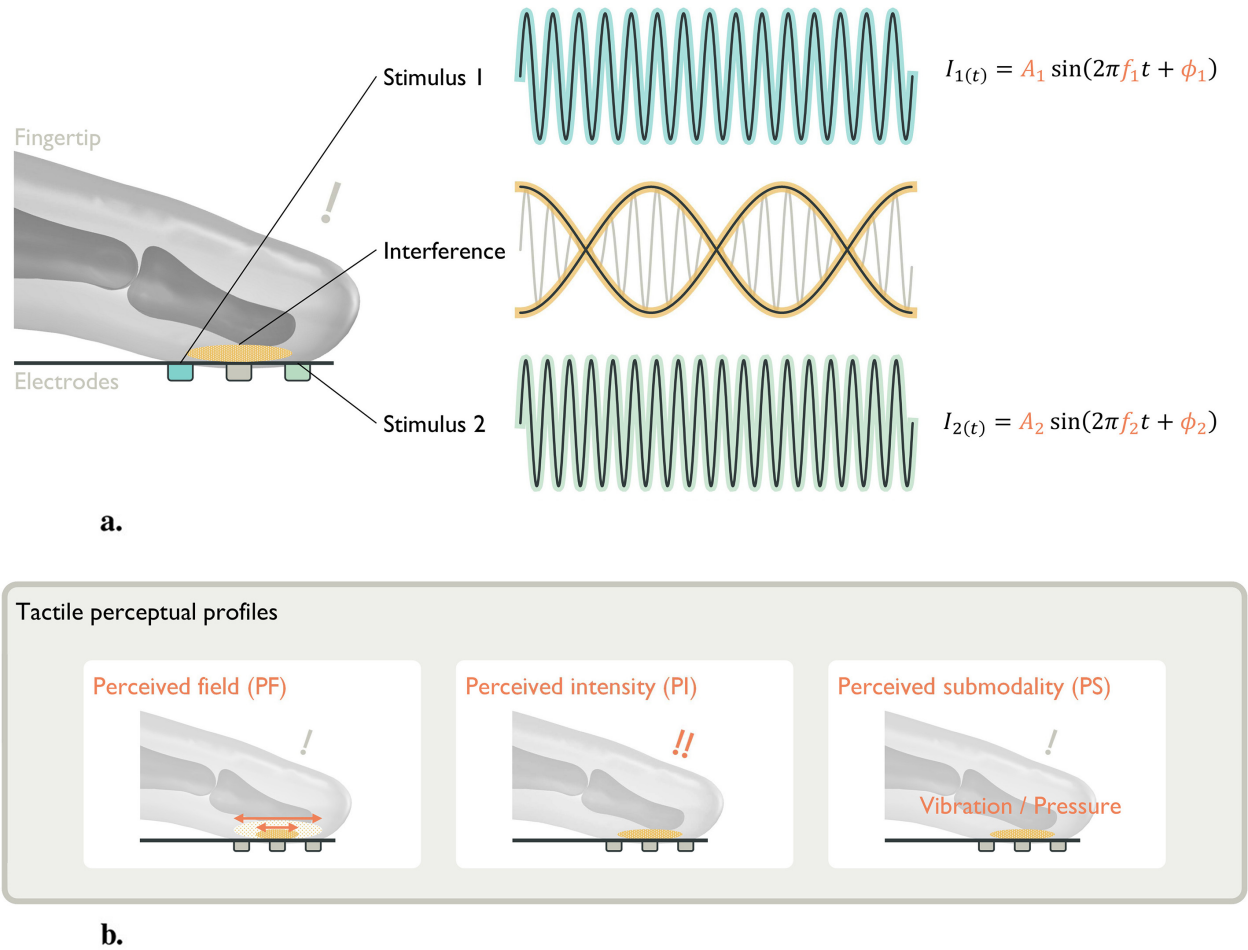
Concept of TIES for presenting tactile sensation: **(a)** Two or more current stimuli, *I*_1_ and *I*_2_, are applied simultaneously (e.g., ϕ_1_ = ϕ_2_) via surface electrodes (blue and green) at kHz frequencies *f*_1_ and *f*_2_, with a slight difference Δ*f* = |*f*_1_−*f*_2_|. These currents generate spatiotemporally oscillating electric fields in the body. The superposition of these fields results in an envelope amplitude that is periodically modulated at Δ*f*. The interference pattern (yellow envelope) can be adjusted by changing the amplitude ratio, frequency, and phase difference. **(b)** Targeting of tactile perceptual profiles. The amplitude ratio, frequency difference and phase difference shape the tactile sensation in terms of spatial extent, intensity, and submodality.

Transcutaneous interferential electrical stimulation (TIES) offers an alternative paradigm. TIES uses two or more high-frequency sinusoidal stimuli (e.g., ≥1 kHz) with slightly different frequencies to create a temporally modulated low-frequency pattern in the target region (Figure 1a). While individual carriers are attenuated by the capacitive properties of neural membranes, the emergent low-frequency beat envelope falls within the physiological response range and can evoke action potentials (APs) in primary afferent fibers. This approach is also called temporal interference (TI) (Guo et al., 2023) and interferential current (IFC) (Goats, 1990; Agharezaee and Mahnam, 2015).

Recently, TIES has been experimentally explored for central nervous system stimulation (Grossman et al., 2017; Mirzakhalili et al., 2020; Gomez-Tames et al., 2021; Wessel et al., 2023) an peripheral nervous system stimulation (Ohara and Hasegawa, 2022; Botzanowski et al., 2022; Lim et al., 2024; Opančar et al., 2025; Jabban et al., 2025). However, establishing a solid theoretical foundation is challenging because TIES generates spatiotemporally interfering electric fields, and axon activation is nonlinear. Hence, computational modeling and simulation play an important role in determining and optimizing stimulus parameters. In principle, three parameters steer the stimulus: amplitude, frequency, and phase. For central nervous models and single-orientation axon models, the amplitude ratio between electrode pairs affects the current density (Grossman et al., 2017) and location of activated axons (Gomez-Tames et al., 2021). Frequency parameters (carrier and beat frequencies) have also been characterized, highlighting that low-frequency modulation is key (Mirzakhalili et al., 2020). Nevertheless, for peripheral nervous and multiple orientation axon models, TIES parameters remain insufficiently characterized. Differences between brain and glabrous skin models lead to different electrode arrangements: two electrode pairs facing each other vs. two electrode pairs aligned along the same axis. Moreover, a few studies have been conducted on tactile perception using computational modeling and simulation. In this study, we operationalize tactile perception in terms of three attributes (PF, PI, and percieved submodality (PS)) and map them to axon activation metrics: PF to the spatial distribution of activated axons, PI to the aggregate of the number of APs, and PS to the anatomical orientations of activated axons (Figure 1b). This study makes the following contributions:

- We provide a systematic characterization of TIES in primary afferent fibers connected to mechanoreceptors (mechanoreceptive fibers), addressing the previously uncharacterized effects of carrier and beat frequencies on the extracellular potential distribution, the activating function, and axon dynamics, based on a simplified three-dimensional computational model and simulations.
- Previous studies typically evaluated only a single orientation, which is insufficient for mechanoreceptive fibers. Here, we systematically characterize three orthogonally oriented axons under identical TIES conditions.
- We derive quantitative guidelines for shaping PF and PI via frequency parameters, thereby reducing reliance on electrode reconfiguration or amplitude modulation and informing the design of compact, programmable electrotactile interfaces.

Building on psychophysical experiments that demonstrate that TIES can evoke tactile sensations (Lim et al., 2024), our preliminary work showed that beat frequency *f*_b_ affects the PF (Ohara et al., 2025). In that study, we examined one TES condition and three TIES conditions, *f*_b_∈{0, 10, 100} Hz, at a carrier frequency of *f*_c_ = 2 kHz with a 1.0 mm axon spacing. Comprehensive characterization requires finer spatial resolution and broader parametric coverage. This study addresses these gaps by evaluating eleven beat frequencies, *f*_b_∈{0, 10, 20, …, 100} Hz, across four carrier frequencies, *f*_c_∈{1, 2, 3, 4} kHz, using the same simulation framework and simplified glabrous skin model with three orthogonally oriented axon arrays at a 0.1 mm spacing. Our results reveal how the carrier and beat frequencies shape the PF and PI, informing design strategies for compact, programmable electrotactile interfaces.

## 2 Methods

### 2.1 Simulation framework

**Figure 2:**
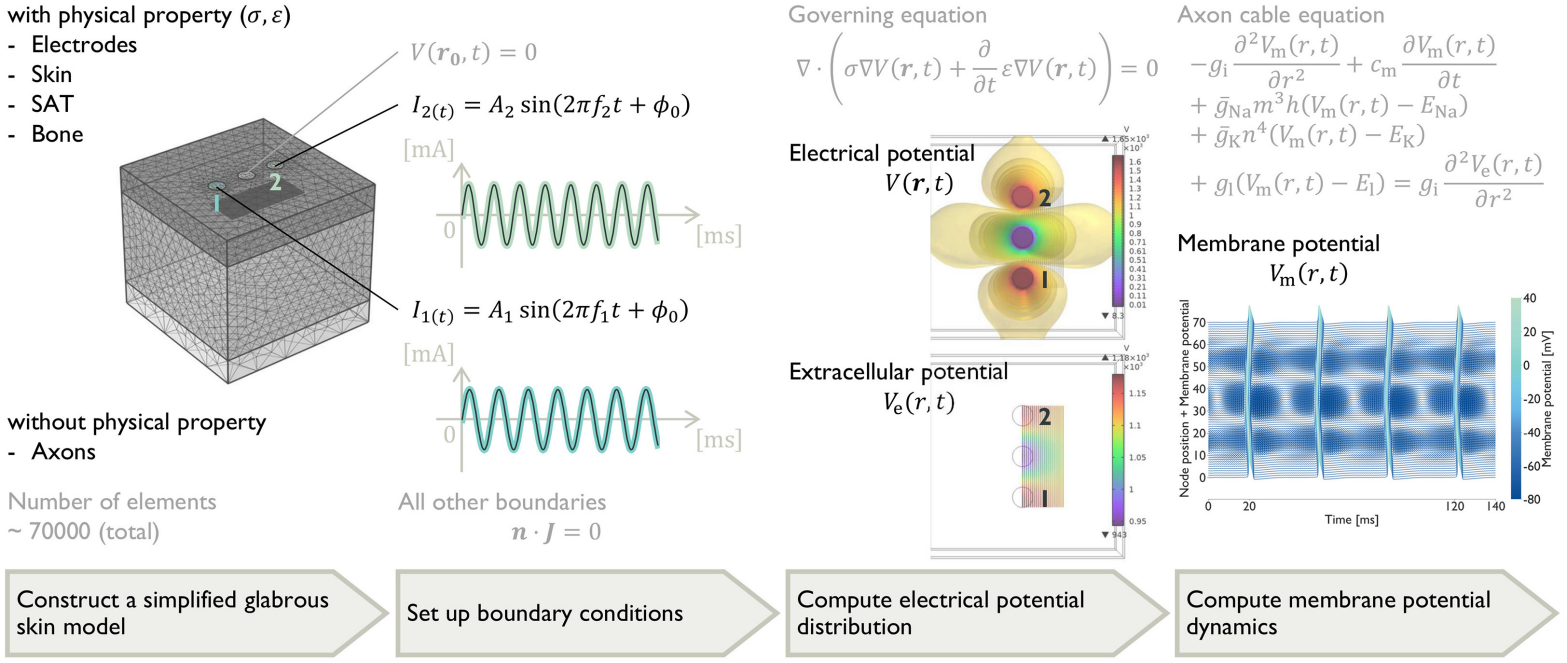
Overview of the simulation framework: Simplified glabrous skin model for FEM simulation, boundary conditions representing TIES, FEM-computed electrical potential distribution across the tissue and along mechanoreceptive fiber trajectories, and axon-scaled simulations of membrane potential dynamics.

We simulated tactile perceptual profiles under TIES by coupling the tissue-scale electrical potential simulation with the axon-scale dynamics simulation. The framework consisted of four steps, following established approaches (Kuhn et al., 2010; Kaihua et al., 2017; Gomez-Tames et al., 2021). First, we constructed a simplified glabrous skin model for finite element method (FEM) simulation. Second, we set up boundary conditions representing TIES stimulation. Third, we computed the electrical potential distribution across the tissue using the FEM and extracted extracellular potentials along the mechanoreceptive fiber trajectories. Fourth, we solved axon cable equations driven by the extracted extracellular potentials to simulate AP initiation and propagation. Finally, we quantified axon activations using metrics aligned with tactile perception: PF and PI. Figure 2 depicts each step: the glabrous skin model, TIES boundary conditions, extracellular potential distribution and axon dynamics.

Although our modeling is simplified, this study focuses on the effects of the stimulation frequency parameters. To reveal the parameter effects while maintaining feasible computation time and cost, we simplified the glabrous skin geometry, tissue heterogeneity, and Aβ fiber properties. Previous studies have shown that the results obtained with simplified models are consistent with psychophysical experiments (Grossman et al., 2017; Kaihua et al., 2017). Thus, this study provides a theoretical foundation for frequency-domain stimulus parameter effects in TIES.

#### 2.1.1 Electrical potential simulation

We solved for the electric potential distribution *V*(***r***, *t*), where ***r*** denotes the spatial position and *t* the time, in the glabrous skin model using three-dimensional FEM. From Maxwell's equations (Ampère–Maxwell's law), the electrical potential distribution is governed by


$$\begin{array}{l} \nabla \cdot \left(\sigma \nabla V\left(r,t\right)+\frac{\partial }{\partial t}\varepsilon \nabla V\left(r,t\right)\right)=0 \end{array}$$ (1)

where σ is the electrical conductivity, ε = ε_0_ε_r_ is the permittivity, with ε_0_ is the vacuum permittivity, and ε_r_ is the relative permittivity. We adopted the frequency-dependent material properties from the IT'IS database (Baumgartner et al., 2025) for each carrier frequency *f*_c_. To preserve consistency between tissue- and axon-scale discretizations, extracellular potentials were obtained by directly sampling *V*(***r***, *t*) at finite-element mesh nodes co-located with axon nodes (without spatial interpolation), where *r* (non-boldface) denotes the axial coordinate along the axon. These time series served as the extracellular drive in the axon dynamics simulations.

#### 2.1.2 Axon dynamics simulation

**Figure 3:**
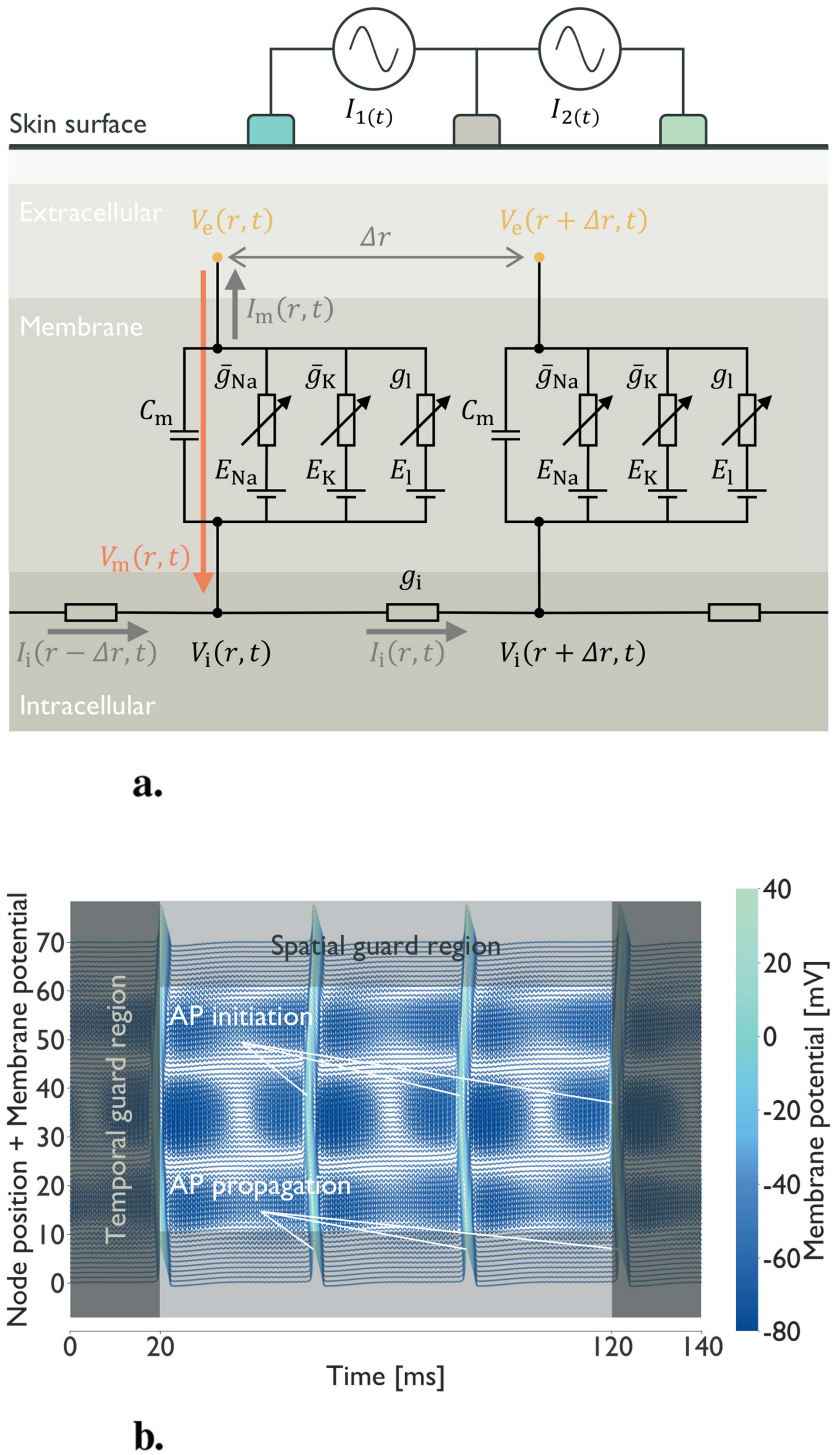
Overview of the axon dynamics simulation: **(a)** Schematic of axon cable equation. The nodal membrane dynamics include channels (sodium (Na), potassium (K), and leak) in parallel with the nodal capacitance (*c*_m_). At the ion channels, ḡ_Na_ and ḡ_K_ denote the maximum conductances, and *E*_Na_ and *E*_K_ are the Nernst potentials. For the leak channel, *g*_l_ is the conductance, and *E*_l_ is the reversal potential. No paranodal, juxtaparanodal, or internodal segments are present. Inside the axon, *I*_i_(*r, t*) is the intracellular current, *V*_i_(*r, t*) is the intracellular potential, and *g*_i_ is the intracellular conductance. At the membrane, *I*_m_(*r, t*) is the membrane current, and *V*_m_(*r, t*) is the membrane potential. Outside the axon, Δ*r* denotes the node-to-node spacing, and *V*_e_(*r, t*) is the extracellular potential at the node interval. **(b)** Visualization of the result from axon dynamics simulation. The example shows a waterfall plot in which traces of membrane potential are vertically offset by node position over the course of the simulation. To count the number of action potentials (APs), temporal and spatial guard regions are used to prevent false positives. Teal undulations crossing nodes indicate AP initiation and propagation. Blue traces indicate the resting state, and their undulations reflect the influence of TIES stimulation.

The extracellular potentials drove the axon-scale simulations of mechanoreceptive fibers. During TIES, the extracellular potential varied spatially along the axon (Figure 3a), creating electrical potential gradients that modulated axon excitability and AP generation. To calculate axon dynamics, we solved the axon cable equation (Equation 2). The equation integrates three models: the cable theory describing an AP propagation in an axon as a passive propagation of charge through an insulated cable (Hermann, 1899; Drukarch et al., 2018), the Hodgkin–Huxley model, which defines the electrical properties of the membrane (Hodgkin and Rushton, 1946; Hodgkin and Huxley, 1952), and McNeal's model, which relates the extracellular potential distribution *V*_e_(*r, t*) to the membrane potential *V*_m_(*r, t*) (McNeal, 1976):


$$\begin{array}{l} \begin{array}{c} -g_{\text{i}}\frac{\partial^{2}V_{\text{m}}\left(r,t\right)}{\partial r^{2}}+c_{\text{m}}\frac{\partial V_{\text{m}}\left(r,t\right)}{\partial t}+{ḡ}_{\text{Na}}m^{3}h\left(V_{\text{m}}\left(r,t\right)-E_{\text{Na}}\right)\\ +{ḡ}_{\text{K}}n^{4}\left(V_{\text{m}}\left(r,t\right)-E_{\text{K}}\right)\\ +g_{\text{l}}\left(V_{\text{m}}\left(r,t\right)-E_{\text{l}}\right)=g_{\text{i}}\frac{\partial^{2}V_{\text{e}}\left(r,t\right)}{\partial r^{2}} \end{array} \end{array}$$ (2)

where *r* denotes the position along the axon, and the *m*, *h*, and *n* are the gating variables. All the parameters, including conductances (ḡ_Na_, ḡ_K_, *g*_l_, *g*_i_), potentials (*E*_Na_, *E*_K_, *E*_l_), membrane capacitance (*c*_m_) and initial gating-variable values, are listed in Table 1 (Hodgkin and Huxley, 1952).

**Table 1:** Parameters of the axon cable equation.

Parameter	Value
Maximum sodium conductance ḡ_Na_	0.12 S/cm^2^
Maximum potassium conductance ḡ_K_	0.036 S/cm^2^
Leakage conductance *g*_l_	0.0003 S/cm^2^
Nernst potential of sodium *E*_Na_	50.0 mV
Nernst potential of potassium *E*_K_	−77.0 mV
Reversal potential of leakage *E*_l_	−54.3 mV
Membrane capacitance *c*_m_	1 μF/cm^2^
Intracellular conductance *g*_i_	0.0282 S/cm
Gating variable *m*	0.00804
Gating variable *h*	0.931
Gating variable *n*	0.129

The left-hand side describes the membrane potential dynamics, whereas the right-hand side, which is proportional to the second spatial derivative of the extracellular potential, is the activating function (AF) (Rattay, 1986). Given the extracellular potentials and appropriate initial conditions, Equation 2 can be numerically integrated to predict membrane potential dynamics and AP generation (Figure 3b).

### 2.2 Model configuration

#### 2.2.1 Simplified glabrous skin model

**Figure 4:**
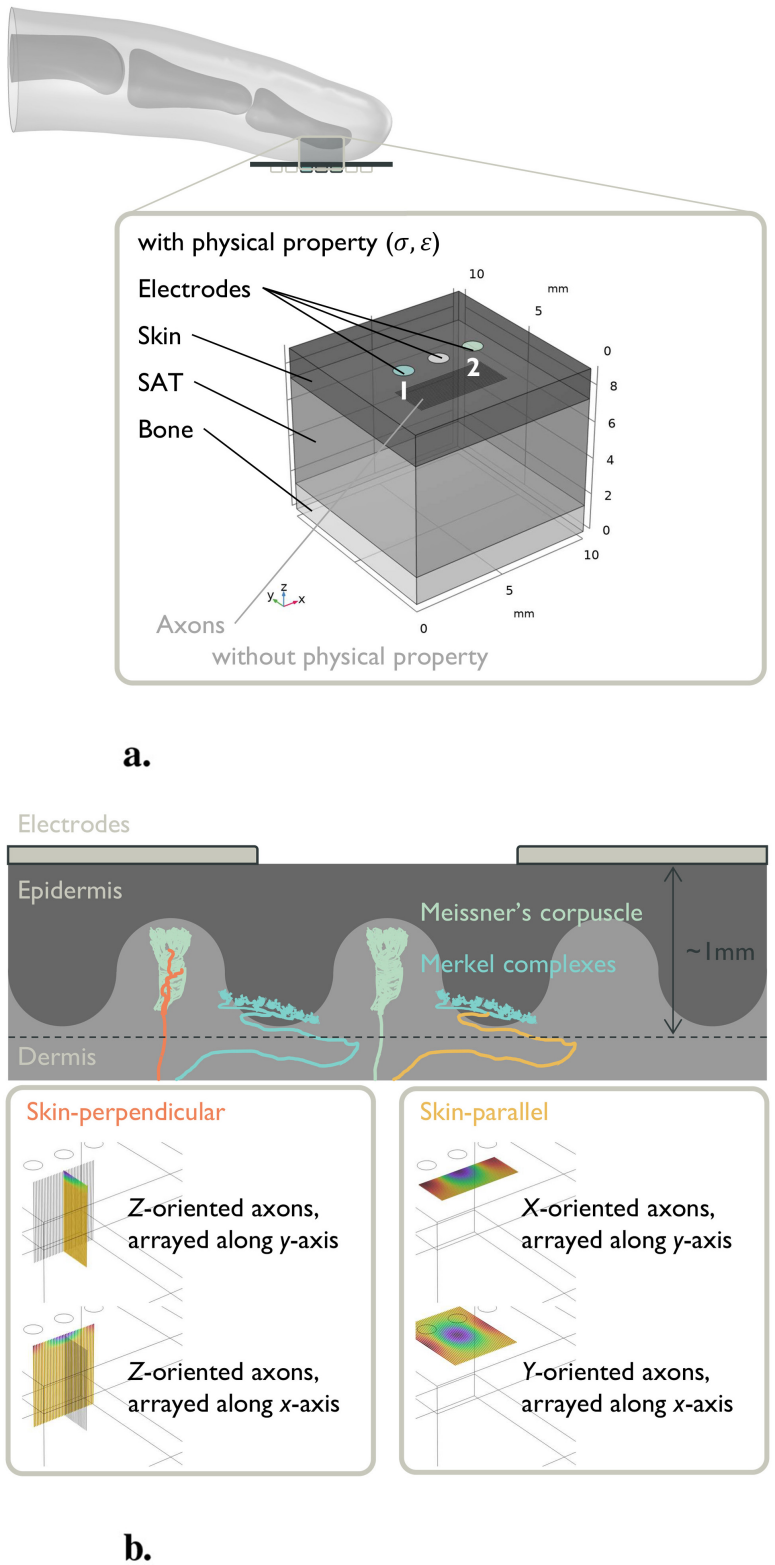
Overview of the computational modeling: **(a)** Simplified glabrous skin model. The upper image is the side view of a fingertip placed on the electrodes. The lower image is an enlarged view of the contact section, where the contact surface is approximated as a plane. **(b)** Simplified axon model of mechanoreceptive fiber in the glabrous skin. The upper illustration depicts the directions of axons connected to Meissner's corpuscles and Merkel complexes. The orange-highlighted line, perpendicular to the skin surface, is the RA1 fiber. The yellow-highlighted line, parallel to the skin surface, is the SA1 fiber. The lower image shows how we modeled these axons. Axons perpendicular to the skin surface are related to RA1 fibers, and those parallel to the skin surface are related to SA1 fibers. Owing to the symmetry of the simplified glabrous skin model, half the number of axons are simulated for the *y*-sweep condition to reduce the computation time.

We constructed a simplified glabrous skin model with a three-layer structure comprising skin, subcutaneous adipose tissue (SAT), cortical bone, and axons (Figure 4a). The computational domain measured 10 mm × 10 mm × 9 mm (width × length × height), with layer thicknesses of 1.6 mm (skin), 5.9 mm (SAT), and 1.5 mm (bone), consistent with previous studies (Serhat et al., 2022; Cei et al., 2025). Electrical properties (σ and ε) were assigned as frequency-dependent functions based on the IT'IS database (Baumgartner et al., 2025). For each tissue layer, σ and ε were interpolated at the *f*_c_. All the values we used are summarized in Tables 2, 3.

**Table 2:** Frequency-dependent electrical properties of glabrous skin tissues.

Tissue	Conductivity (mS/m)				Relative permittivity (× 10^3^)			
	Carrier frequency (kHz)				Carrier frequency (kHz)			
	1	2	3	4	1	2	3	4
Skin	0.200	0.200	0.200	0.201	1.14	1.14	1.14	1.13
SAT	42.7	42.5	42.3	41.7	19.3	8.26	4.97	3.28
Bone (cortical)	20.2	20.2	20.3	20.3	2.70	1.70	1.25	0.995

**Table 3:** Geometry of simplified glabrous skin and axon models.

Parameter	Value
*Simplified glabrous skin model*	
Domain size	10 mm × 10 mm × 9 mm
Skin thickness	1.6 mm
SAT thickness	5.9 mm
Bone thickness	1.5 mm
Electrode diameter	1 mm
Electrode spacing (center-to-center)	2 mm
Axon lateral spacing	0.1 mm
15.6-7.8,-1.3242ptSampling node spacing along axon Δ*r*	0.1 mm
*Axon model*	
Length *L*_axon_	5 mm
Diameter	8.7 μm
Node-to-node spacing Δ*L*_axon_	0.1 mm

To realize TIES with a minimal electrode configuration, we placed three circular surface electrodes (diameter: 1 mm) in a straight line with a center-to-center spacing of 2 mm on the skin surface. The diameter was consistent with previous studies, which used electrodes ranging from 0.5 mm (Botzanowski et al., 2022; Lim et al., 2024) to 3.0 mm (Lin et al., 2022). We implemented two interferential pairs that shared the center electrode, yielding the smallest possible pair separation. This shared electrode geometry produces a well-defined focal region of interference (Botzanowski et al., 2022).

In the electrical potential simulation, each axon was represented as a straight *L*_axon_ = 5 mm path with 51 equally spaced nodes (Δ*L*_axon_ = Δ*r* = 0.1 mm) within a central subvolume of 5 mm × 5 mm × 5 mm (0.1 mm lateral spacing between neighboring axons) as shown in Figure 4b. The axon volume was negligible relative to the tissue, and thus, was not assigned electrical properties.

#### 2.2.2 Simplified axon model of mechanoreceptive fiber

Tactile sensations are conveyed by Aβ fibers innervating four mechanoreceptor classes (Kandel et al., 2021; Deflorio et al., 2022). Type 1 endings comprise Meissner's corpuscles (RA1, rapidly adapting type 1) and Merkel complexes (SA1, slowly adapting type 1), which are located near the epidermis (0.5–1.0 mm from the skin surface) and have small receptive fields. Type 2 endings comprise Pacinian corpuscles (RA2) and Ruffini endings (SA2), which are located deeper in the dermis (2.0–3.0 mm from the skin surface) and exhibit large receptive fields. RA fibers (RA1/RA2) respond to dynamic stimuli such as vibration, slip and fine texture, whereas SA fibers (SA1/SA2) encode sustained pressure, shape, and deformation. Consistent with anatomical observations, RA1-associated fibers tend to approach the epidermis approximately perpendicular to the skin surface, whereas SA1-associated fibers frequently run parallel to the skin surface (Provitera et al., 2007). In contrast, the myelinated endings of RA2 and SA2 run parallel to the skin surface within the same dermal layer. Unlike RA1 and SA1, which can be distinguished by their orientations, faithfully simulating RA2 and SA2 would require receptor-specific geometric and electrical parameters beyond axon orientation, which are beyond the scope of this paper. Guided by these observations and our focus on PF and PI, we instantiated mechanoreceptive fiber trajectories with three orthogonal orientations at 1 mm depth:

- **X-oriented axons**: parallel to the skin surface, running along the *x*-axis (i.e., the row of three-electrodes) and arrayed along the *y*-axis;
- **Y-oriented axons**: parallel to the skin surface, running along the *y*-axis and arrayed along the *x*-axis;
- **Z-oriented** **(*y*)** **axons**: perpendicular to the skin surface, running along the *z*-axis, and arrayed along the *y*-axis;
- **Z-oriented** **(*x*)** **axons**: perpendicular to the skin surface, running along the *z*-axis, and arrayed along the *x*-axis.

Henceforth, we refer to *x*- and *y*-oriented axons as skin-parallel axons and *z*-oriented axons as skin-perpendicular axons.

Although Aβ fibers are myelinated, our objective was to test whether spatiotemporal variations in the *V*_e_(*r, t*) generated by TIES are sufficient to elicit APs. Accordingly, each mechanoreceptive fiber was modeled as a continuous cable governed by the axon cable equation. The axon diameter of RA1 fiber ranges from 7 to 11 μm, and that of SA1 fiber ranges from 6 to 12 μm (Kandel et al., 2021). Considering typical Aβ fiber diameters (McIntyre et al., 2002; Kaihua et al., 2017), we set the axon diameter to 8.7 μm to represent them. In glabrous skin, C fibers are also related to tactile perception such as mechanical, thermal, and nociceptive sensations. Their axon diameters range from 0.2 to 1.5 μm. Because Aβ fibers have lower axial resistivity owing to their larger diameter, they are more easily activated than C fibers. Consequently, we focused on Aβ fibers and did not model C fibers in this study.

### 2.3 Stimulus parameters

#### 2.3.1 Boundary conditions

In the glabrous skin model, the central electrode was grounded (Dirichlet: *V* = 0), and the two flanking electrodes delivered the stimulation currents under current-controlled boundary conditions. All other external boundaries were treated as electrically insulating (***n***·***J*** = 0), where ***n*** is the unit normal vector, and ***J*** is the current density vector, yielding a well-posed boundary-value problem.

To simulate TIES, the two stimulation electrodes delivered sinusoidal currents at slightly different frequencies, *f*_1_ = *f*_c_ and *f*_2_ = *f*_c_+*f*_b_:


$$\{\begin{array}{l} I_{1}(t):\;=A_{1}\sin(2\pi f_{1}t+\phi_{1})\\ I_{2}(t):\;=A_{2}\sin(2\pi f_{2}t+\phi_{2}) \end{array}$$ (3)

with amplitude *A*_1_ = *A*_2_ = 0.5 mA per electrode (total applied current 1 mA) and phase ϕ_1_ = ϕ_2_ = 0 rad. Unless otherwise noted, currents were in phase, *f*_b_≪*f*_c_, and material properties were evaluated at each *f*_c_. We examined carrier frequencies *f*_c_∈{1, 2, 3, 4} kHz and beat frequencies *f*_b_∈{0, 10, 20, …, 100} Hz (10 Hz steps). These ranges were selected based on previous experimental results: perceptible tactile sensations via TIES have been reported at *f*_c_ = 1 kHz for *f*_b_≥10 Hz (tested *f*_b_ = {0, 10, 50, 100} Hz) (Lim et al., 2024), and motor responses in mice elicited by TIES targeting the central nervous system have been demonstrated for *f*_c_ up to 4 kHz (tested *f*_c_ = {1, 2, 3, 4, 5} kHz) (Grossman et al., 2017).

#### 2.3.2 Amplitude normalization

To isolate the effects of *f*_b_ and enable fair comparison across *f*_c_ and axon orientations *r*∈{*x, y, z*}, we normalized stimulus amplitude based on a reference PI criterion prior to evaluation. Normalization was performed for three reference axons (one per orientation) across four carrier frequencies, yielding 3 × 4 = 12 conditions. The reference axons passed through (*x, y, z*) = (5.0, 5.0, 8.0) in the glabrous skin model, which was beneath the center of the electrode array, and aligned along one of the *x*-, *y*-, or *z*-axes.

For each condition (*f*_c_, *f*_b_) and axon orientation, we determined the scaling factor *S*(*f*_c_, *f*_b_) such that exactly *N*_AP, ref_ = *f*_b_*T*_w_ APs were detected in the reference axon during the evaluation window *T*_w_. The scaling factor was defined as the minimum gain satisfying the constraint


$$\begin{array}{c} S\left(f_{\text{c}},f_{\text{b}}\right):=\min\left\{k\in \left[10^{-4},10^{1}\right]|N_{\text{AP}}\left(k;f_{\text{c}},f_{\text{b}}\right)\geq N_{\text{AP},\text{ref}}\right\} \end{array}$$ (4)

where *N*_AP_(*k*; *f*_c_, *f*_b_) denotes the number of APs detected in the reference axon during *T*_w_ under stimulus gain *k* given (*f*_c_, *f*_b_). In this study, we set *f*_b_ = 30 Hz and *T*_w_ = 0.1 s, yielding *N*_AP, ref_ = 3. *S*(*f*_c_, *f*_b_) was computed using binary search with a tolerance of 0.1% for *k*. The upper limit was set because TIES current densities above 0.05 A/mm elicited only pain perception (Lim et al., 2024).

All subsequent evaluations used applied current amplitude scaled by *S*(*f*_c_, *f*_b_ = 30) such that, under the same *f*_c_, the number of APs at the reference axon matched *N*_AP, ref_ at the reference *f*_b_. This enabled a comparison of PF and PI across different *f*_b_.

### 2.4 Evaluation

Under each condition (*f*_c_, *f*_b_) and axon orientation, we quantified PF and PI using axon activation metrics. PF captures the spatial extent of the perception and PI its strength. Orientation-specific recruitment contextualizes PS. Let *N*_AP_(*i*) denote the number of APs of axon *i* running along the *x*-, *y*-, or *z*-axis, within the evaluation time window. Contiguity was defined for adjacent axon positions at 0.1 mm spacing.

#### 2.4.1 Extracellular potential

We solved for *V*(***r***, *t*) in the glabrous skin model and sampled *V*_e_(*r, t*) along axon pathways to characterize the spatiotemporal field structure. Because the AF is proportional to $\partial^{2}V_{\text{e}}\left(r,t\right)/\partial r^{2}$, to characterize the *f*_c_ dependence, we computed the RMS value of *V*_e_(*r, t*) at each node position *r*_*i*_ as follows:


$$\begin{array}{c} V_{\text{e},\text{RMS}}:=\sqrt{\frac{1}{T_{\text{w}}}\overset{T_{\text{w}}}{\underset{t=0}{\sum }}{\left(V_{\text{e}}\left(r,t\right)\right)}^{2}}. \end{array}$$ (5)

To isolate the effect of *f*_b_ and enable fair comparison across *f*_c_, we normalized the AF for each axon orientation and each *f*_c_ condition. Specifically, we analyzed the maximum activating function over the evaluation time window, defined as


$$\begin{array}{c} AF_{\max}:=\underset{t\in \left[0,T_{\text{w}}\right]}{\max}|\frac{\partial^{2}V_{\text{e}}\left(r,t\right)}{\partial r^{2}}|. \end{array}$$ (6)

#### 2.4.2 Tactile perceptual metrics

From a neural coding perspective, tactile perception can be decomposed into PF, which is related to the receptive field, PI, and PS, which depends on the mechanoreceptor type and activation pattern (Muniak et al., 2007; Kandel et al., 2021; Deflorio et al., 2022). In this study, we quantified PF using the spatial distribution of the activated axons and PI using the population of APs, and we contextualized PS by comparing orientation-specific recruitment across the three axon orientations.

An AP was counted for axon *i* if (i) its initiation time *t*_*k*_∈[*t*_0_, *t*_0_+*T*_w_], and (ii) it successfully propagated to reach either of the spatial guard region boundaries (distance *L*_guard_ from the evaluation region) during *T*_total_. APs initiating within the temporal or spatial guard regions were excluded. Activated axons were defined as those generating at least one AP: $A:=\left\{i:N_{\text{AP}}\left(i\right)\geq 1\right\}$. We defined the activation threshold as $N_{\text{AP},\text{thr}}:=|A{|}^{-1}\underset{i\in A}{\sum }N_{\text{AP}}\left(i\right)$, identified all contiguous regions among activated axons that exceeded this threshold,


$$\begin{array}{c} R:=\left\{i\in A|N_{\text{AP}}\left(i\right)\geq N_{\text{AP},\text{thr}}\right\} \end{array}$$ (7)

and selected the region containing the global maximum firing rate *N*_AP, max_(*i*) as the primary region R. If multiple regions existed, we reported the longest as the primary region and noted the secondary peaks as potential multiple location perception.

We quantified PF and PI based on the spatial distribution and number of APs. Based on previous TIES simulations in the central nervous system (Gomez-Tames et al., 2021), we defined PF as the spatial length of the primary region R:


$$\begin{array}{c} PF:=x_{\max}\left(R\right)-x_{\min}\left(R\right) \end{array}$$ (8)

where *x*_max_ and *x*_min_ are the maximum and minimum positions of activated axons within R. This metric quantified the spatial extent of the perception. Assuming that PI correlates with the total neural activity across activated axons (Mountcastle et al., 1966; Muniak et al., 2007), we defined PI as the population of APs within R:


$$\begin{array}{c} PI:=\underset{i\in R}{\sum }N_{\text{AP}}\left(i\right) \end{array}$$ (9)

### 2.5 Simulation configulation

We used COMSOL Multiphysics (version 6.1) for the electrical potential simulation and Python (version 3.11.9) with NEURON (version 8.2.6) for the axon dynamics simulation. This coupling constituted the simulation framework required for systematic TIES characterization. The electrical potential simulation was performed on a workstation (Intel Xeon W-2223, 4 cores, 64 GB RAM), requiring approximately 40 h per *f*_c_ condition for each axon orientation (mesh: ~700 K elements). The axon dynamics simulation was performed using a laptop (Intel Core i5-1345U, 10 cores, 16 GB RAM), requiring approximately 12 h per *f*_c_ condition with 4-core MPI. The total sequential execution time was approximately 528 h.

Throughout the simulation framework, we employed a synchronized time step of Δ*t* = 10 μs for both the electrical potential and axon dynamics simulations. This choice provides sufficient temporal resolution across all the tested TIES conditions. For the highest carrier frequency (4 kHz), the Nyquist criterion requires sampling at least every 125 μs. Our choice of a 10 μs step thus affords 12.5 × oversampling.

For the axon dynamics simulations, all analyses were performed over a fixed window [*t*_0_, *t*_0_+*T*_w_] with temporal guard regions of duration *T*_guard_ appended before and after the window (*t*_0_ = *T*_guard_). Spatial guard regions *L*_guard_ were placed at both ends of each axon pathway to prevent edge effects. Guard regions were excluded from all analyses. We used *T*_guard_ = 20 ms (total simulation time for each axon: *T*_total_ = 2 × *T*_guard_+*T*_w_ = 140 ms) and *L*_guard_ = 1.0 mm.

We addressed two methodological aspects: suppression of stimulus onset/offset artifacts and AP detection. To suppress onset/offset artifacts, we applied periodic wrapping to the temporal guard regions. Because the *T*_*w*_ = 100 ms was periodic for all tested frequency combinations, signals immediately before *t*_0_ and after *t*_0_+*T*_w_ were concatenated to, establish steady-state conditions at the simulation onset. For AP detection, an AP was counted when the membrane potential crossed the threshold *V*_m, thr_ = −20 mV with a positive slope within the evaluation time window, using the standard function implemented in NEURON.

## 3 Results

### 3.1 Extracellular potential

**Figure 5:**
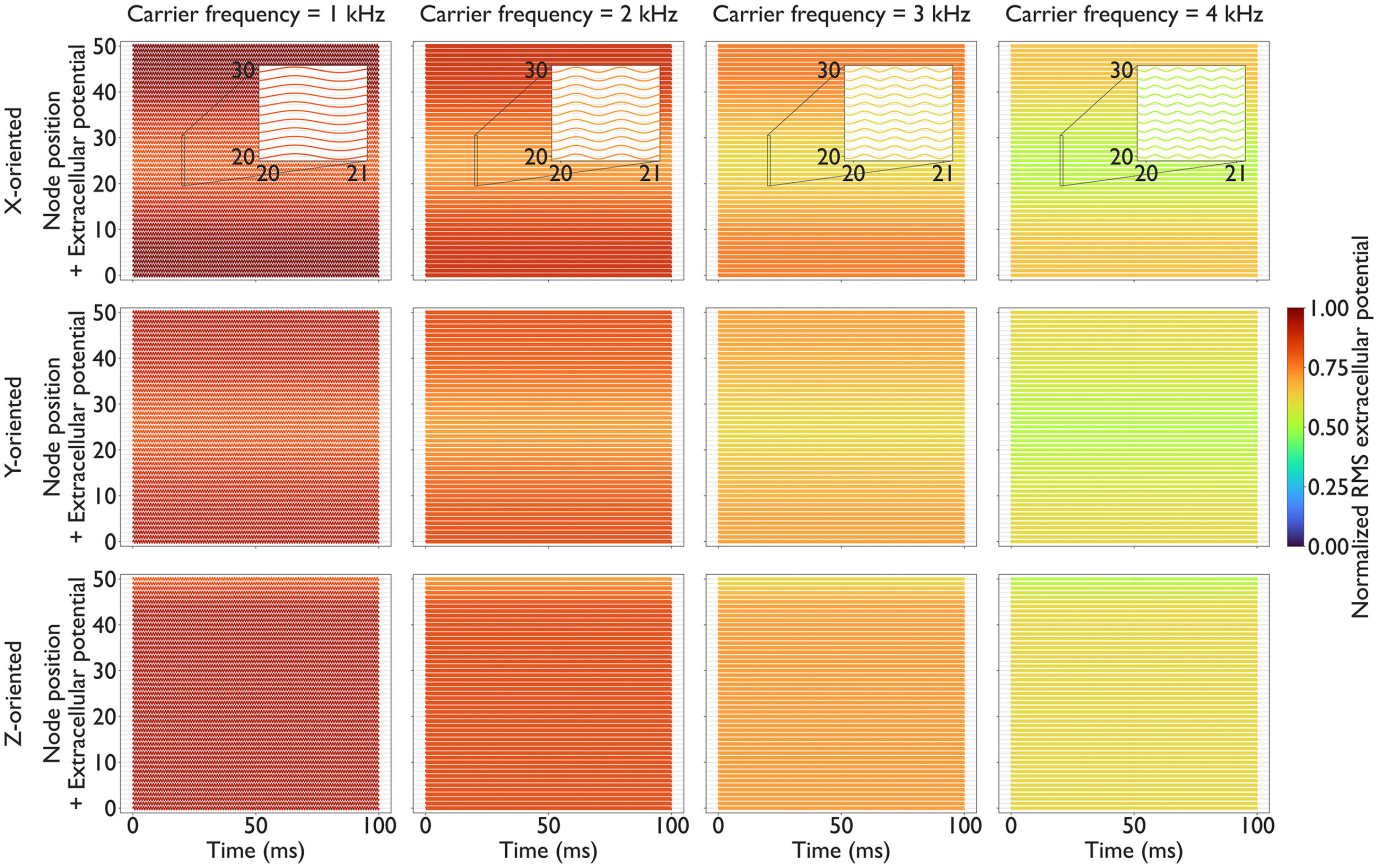
Carrier frequency *f*_c_ effects on extracellular potential *V*_e_(*r, t*) along the reference axons: Each panel shows a waterfall plot in which traces of *V*_e_(*r, t*) [mV] are vertically offset according to node position along the axon over the course of the simulation. The vertical offset corresponds to the internodal spacing of 0.1 mm. Each plot shows RMS values calculated using Equation 5 and normalized across all panels (on a 0–1 scale). Columns correspond to increasing *f*_c_∈{1, 2, 3, 4} kHz with *f*_b_ = 0 Hz. Rows correspond to axon orientation. Colors indicate the RMS *V*_e_(*r, t*) normalized to the global maximum across all panels (0–1 scale). Enlarged views highlight the differences in the effects of *f*_c_ in the middle of the axon (node 20–30) over 1 ms.

**Figure 6:**
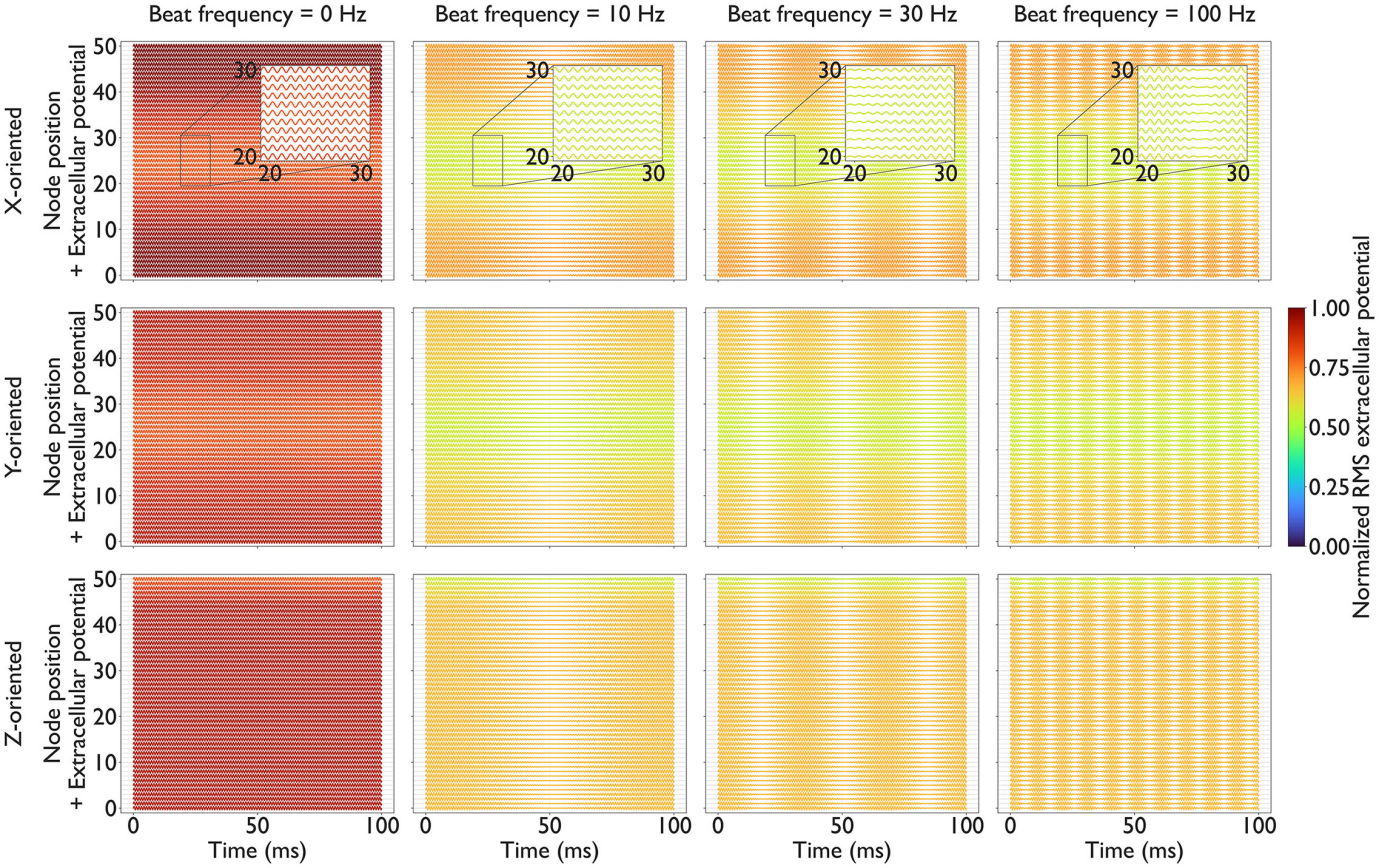
Beat frequency *f*_b_ effects on extracellular potential *V*_e_(*r, t*) along the reference axons: Each panel shows a waterfall plot in which traces of *V*_e_(*r, t*) [mV] are vertically offset according to node position along the axon over the course of the simulation. Each plot shows RMS values calculated using Equation 5 and normalized across all panels. Columns correspond to four beat frequencies: *f*_b_ = 0 Hz (minimum), 10 Hz (minimum for beat generation), 30 Hz (used in axon dynamics simulation), and 100 Hz (maximum) with a carrier frequency of *f*_c_ = 1 kHz. Rows correspond to axon orientation. Colors indicate the RMS the *V*_e_(*r, t*), normalized to the global maximum across all panels (0–1 scale). Enlarged views highlight differences in the effects of *f*_b_ in the middle of the axon (node 20–30) over 10 ms.

Figures 5, 6 depict extracellular potentials along the reference axons. Across all conditions, *f*_c_ exerted a dominant influence on the RMS amplitude of the extracellular potentials, and *f*_b_ produced temporal interference patterns. Increasing *f*_c_ from 1 to 4 kHz reduced the RMS amplitude of the extracellular potentials by approximately a factor of two for all axon orientations. In contrast, *f*_b_ had a minor effect on the RMS amplitude. *f*_b_ = 0 Hz produced almost twice the RMS value compared to that obtained using *f*_b_∈{10, 30, 100} Hz. Spatially, the extracellular potential of the reference axons was the smallest beneath the grounded electrode. These results indicate that higher carrier frequencies attenuate the extracellular potentials along mechanoreceptive fibers due to frequency-dependent tissue impedance.

#### 3.1.1 Activating function

**Figure 7:**
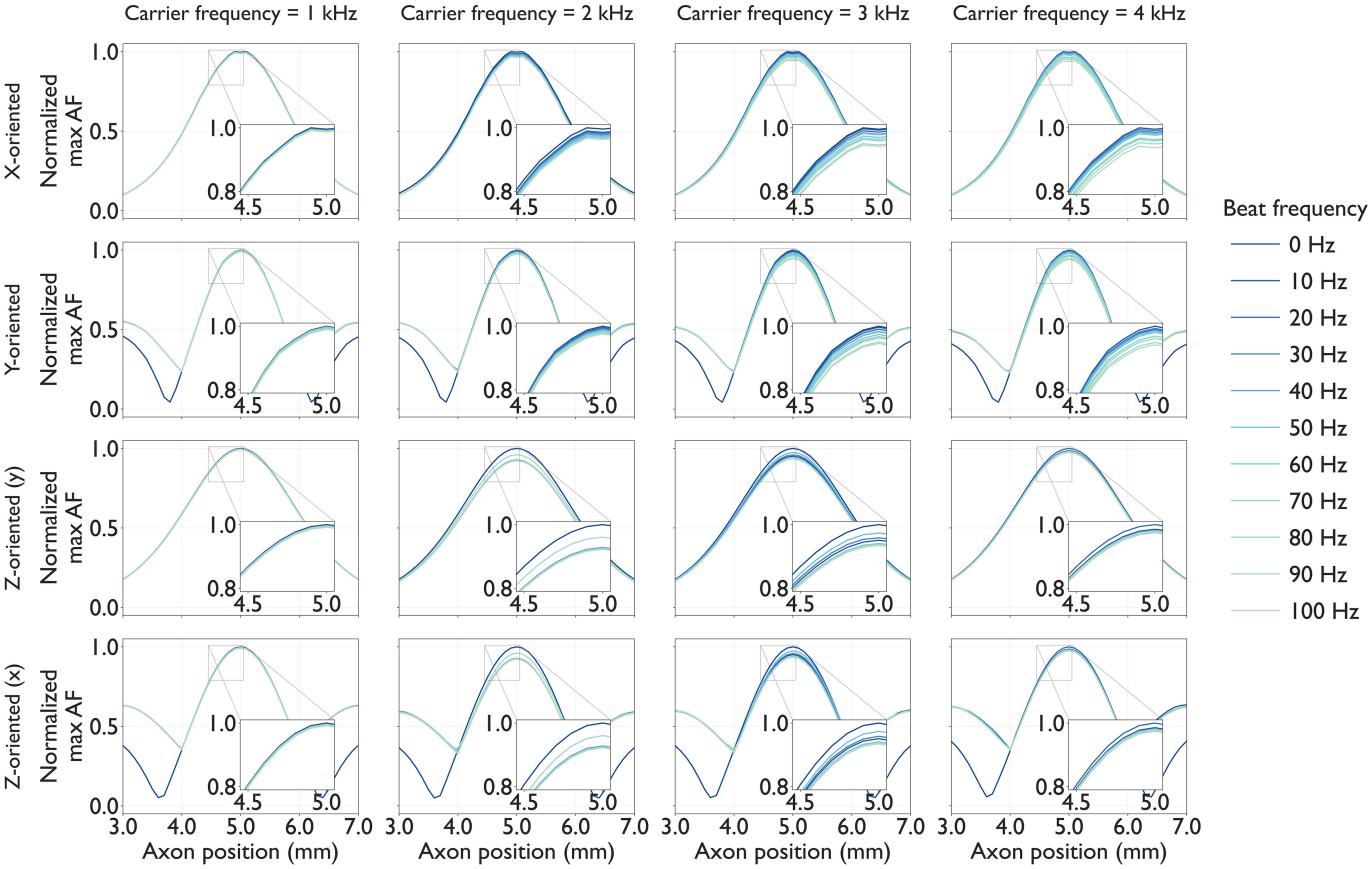
Normalized maximum activating function (AF_max_) for each axon: Each panel shows a line plot in which traces of AF_max_ along each axon over the course of the simulation, and values are normalized to the maximum within each panel (0–1 scale). Each plot represents a different beat frequency *f*_b_∈{0, 10, 20, …, 100} Hz. Columns correspond to four carrier frequencies *f*_c_∈{1, 2, 3, 4} kHz. Rows correspond to axon orientation. Enlarged views highlight a slight difference in AF_max_ near the center of electrode array (across five axons).

Figure 7 summarizes the results for AF_max_ normalized for each condition (panel), highlighting the spatial modulation patterns. Because increasing *f*_c_ from 1 to 4 kHz reduces the extracellular potentials by approximately half, we anticipated that the relative differences associated with *f*_b_, which only slightly affects the RMS amplitude, would be more apparent in the normalized AF_max_. If differences exist within the minor-effect range of *f*_b_, they could potentially be leveraged to shape the tactile perception via TIES.

Across all conditions, as expected, the range of normalized AF_max_ increased with *f*_c_ at the reference axons. For a given condition, increasing *f*_b_ reduced AF_max_. The normalized AF_max_ profiles revealed that carrier frequencies of approximately *f*_c_ = 3 kHz provided the greatest sensitivity to *f*_b_ adjustment. For skin-parallel axons, modulation by *f*_b_ was most pronounced at *f*_c_∈[3, 4] kHz, whereas for skin-perpendicular axons, *f*_b_ driven changes in AF_max_ were the largest at *f*_c_ = 2 or 3 kHz.

Spatially, the distribution of AF_max_ depended on the sweep direction. *x*-oriented axons and *z*-oriented axons arrayed along *y*-axis (*x* = 5.0) exhibited predominantly unimodal profiles, with peak AF_max_ concentrated near the center beneath the grounded electrode. In contrast, *y*-oriented axons and *z*-oriented axons arrayed along *x*-axis (*y* = 5.0) showed trimodal AF_max_ distributions: the strongest peak appeared beneath the grounded electrode, with two symmetric secondary peaks located beneath each stimulation electrode. These trimodal activation patterns suggest the potential for recruiting axons at three spatially distinct locations, which may correspond to multiple location tactile perception under sufficiently high stimulus amplitudes.

### 3.2 Axon dynamics

**Figure 8:**
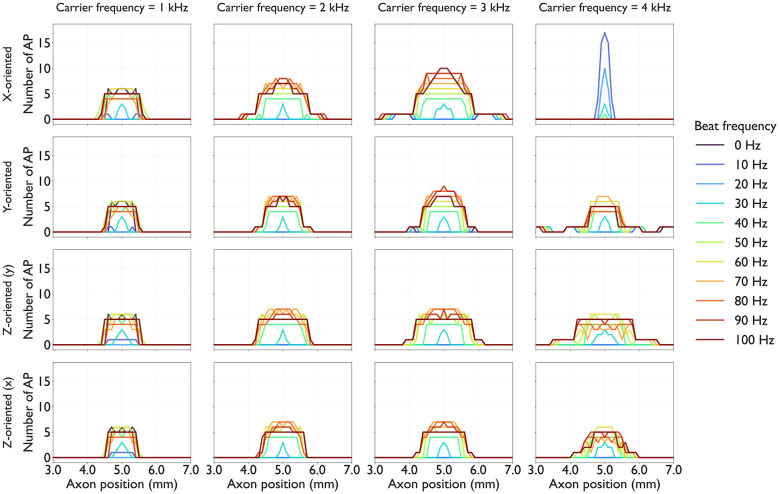
Number of action potentials (APs) of each axon: Each panel shows a line plot in which traces of AP along each axon over the course of the simulation, and stimulus amplitude normalization using scaling factors *S*(*f*_c_, *f*_b_ = 30) was applied within each panel. Each plot represents a different beat frequency *f*_b_∈{0, 10, 20, …, 100} Hz. Columns correspond to four carrier frequencies *f*_c_∈{1, 2, 3, 4} kHz. Rows correspond to the axon orientation.

By substituting the extracellular potentials into the axon dynamics simulation, Figure 8 shows the spatial distribution of activated axons and the number of APs elicited in each axon.

**Figure 9:**
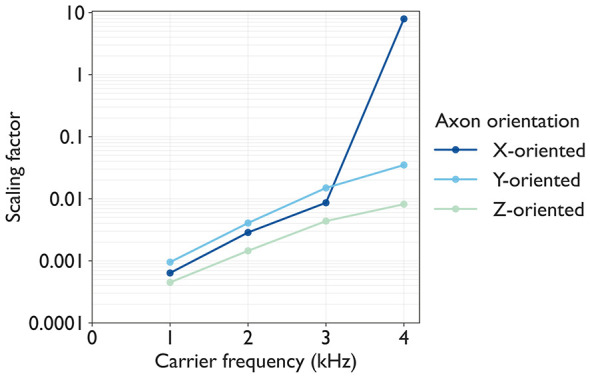
Scaling factors *S*(*f*_c_, *f*_b_) computed for the reference axons at *f*_b_ = 30 Hz: Each plot represents a different axon orientation, and each dot represents *S*(*f*_c_, *f*_b_) at the condition (*f*_c_, *f*_b_ = 30 Hz) to evoke targeted number of action potentials *N*_AP, ref_ = 3.

The computed scaling factors *S*(*f*_c_, *f*_b_ = 30) for all conditions are plotted in Figure 9. We observed that higher *N*_AP, ref_ values required larger scaling factors. As *f*_c_ increased from 1 to 4 kHz, *S*(*f*_c_, *f*_b_ = 30) increased exponentially.

#### 3.2.1 Perceived field

**Figure 10:**
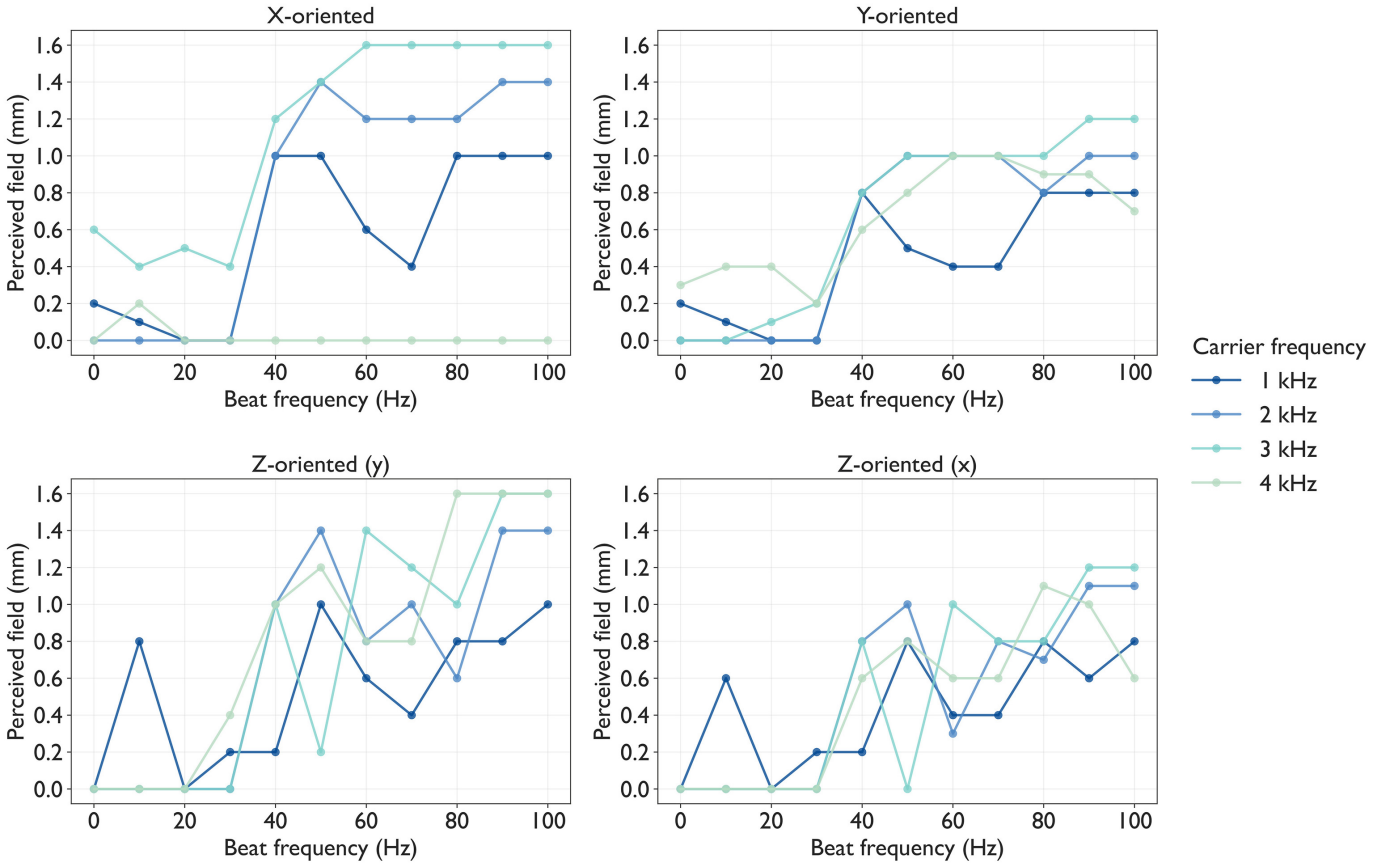
Frequency effects on spatial length of the primary region R (PF, perceived field) via TIES: Each panel shows a line plot in which computed from Figure 8 using Equation 8. Each plot represents a different carrier frequency *f*_c_∈{1, 2, 3, 4} kHz, and each dot represents the PF value at the condition (*f*_c_, *f*_b_). Each panel corresponds to the axon orientation.

From the results shown in Figure 8, we computed the primary region R and summarized the PF in all conditions, as shown in Figure 10. The PF value increased as *f*_b_ increased from 30 to 100 Hz across all conditions except for *x*-oriented axon at 4 kHz carrier frequnecy. Moreover, the PF at *f*_b_≥40 Hz typically exceeded that at *f*_b_ = 0 Hz, demonstrating the spatial spreading effect of *f*_b_ on the recruitment of activated axons. Across all axon orientations, PF was the largest at *f*_c_ = 2 or 3 kHz. For example, at *f*_c_ = 3 kHz, the range of PF when *f*_b_ was changed across all axon orientations was more than 1.0 mm, which corresponds to the electrode diameter. This indicates that PF can be adjusted over a spatial range exceeding the electrode diameter under a given condition (*f*_c_, *f*_b_).

#### 3.2.2 Perceived intensity

**Figure 11:**
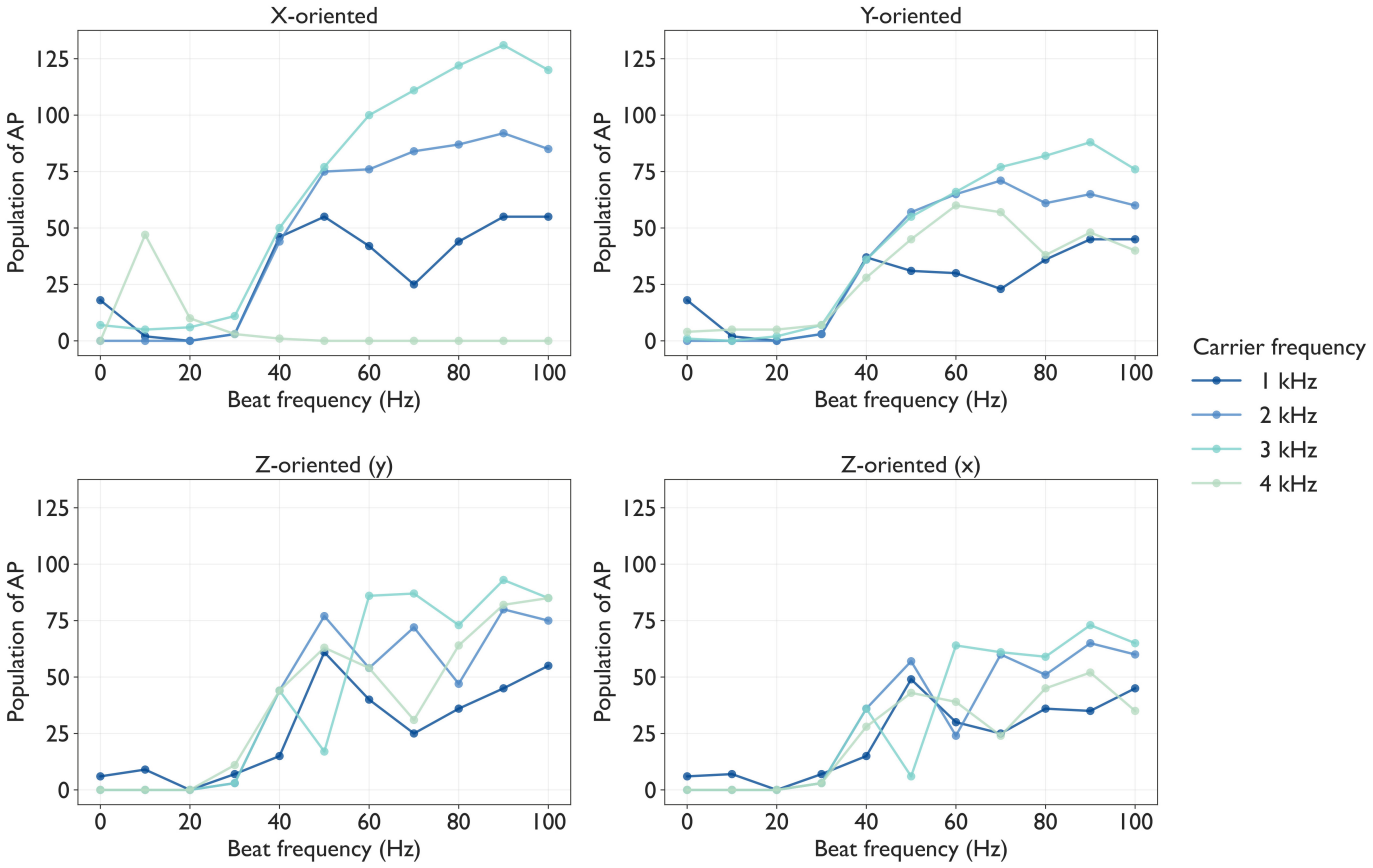
Frequency effects on population of action potentials (APs) of the primary region R (PI, perceived intensity) via TIES: Each panel shows a line plot in which computed from Figure 8 using Equation 9. Each plot represents a different carrier frequency *f*_c_∈{1, 2, 3, 4} kHz, and each dot represents PI value at the condition (*f*_c_, *f*_b_). Each panel correspond to axon orientation.

Using the same primary region R as the PF, we evaluated PI as the total number of APs. Figure 11 shows the PI for all conditions. The overall trend of PI as a function of *f*_b_ resembled that of PF, exhibiting a systematic increase with increasing *f*_b_. This implies that PF and PI depend on the frequency parameters of TIES. However, the *f*_c_ effects were more pronounced for PI than for PF: *f*_c_ = 2 or 3 kHz values consistently yielded a higher PI than other values (*f*_c_ = 1 or 4 kHz) at the same *f*_b_. This indicates that PI can be adjusted through *f*_c_ selection.

## 4 Discussion

This paper presents a simplified computational model to systematically examine how carrier and beat frequencies of TIES shape tactile perceptual profiles. We coupled electrical potential simulations with axon dynamics and quantified the scaling factors *S*(*f*_c_, *f*_b_), AF_max_, and AP counts across three orthogonal axon orientations. Our main findings are threefold. First, increasing carrier frequency from 1 to 4 kHz requires an exponentially larger stimulus amplitude (scaling factors) to elicit the target number of APs at the reference axons. Second, the carrier frequency sets the upper bounds for PF and PI, whereas the beat frequency adjusts the PF and PI within these bounds. Third, orientation-specific activation follows the order *z*-oriented>*x*-oriented>*y*-oriented, suggesting the preferential activation of skin-perpendicular axons, consistent with vibratory perception. Our simulation framework couples two components that can be validated separately: tissue-scale electrical potential distribution and axon-scale dynamics.

As the carrier frequency increases from 1 to 4 kHz, the tissue properties change with frequency. Skin conductivity remains nearly constant, but the effective admittance $|\sigma^{*}|=\sqrt{\sigma^{2}+{\left(2\pi f\varepsilon \right)}^{2}}$ increases by approximately 50% as the capacitive term 2π*fε* becomes significant at kHz frequencies. In contrast, SAT exhibits decreasing conductivity and permittivity, resulting in nearly constant admittance across 1–4 kHz.

Under current-controlled conditions, the RMS extracellular potential decreases as *f*_c_ increases, reflecting the increased effective admittance of the composite tissue. Furthermore, the frequency-dependent increase in skin admittance causes a greater proportion of current to flow through the skin layer rather than deeper tissues. These phenomena were reproduced in Figures 5, 6.

Regarding the beat frequency, changing *f*_b_ slightly alters AF_max_ for both skin-parallel and skin-perpendicular axons (Figure 7). Although the change in AF_max_ is modest, a consistent decrease is observed as *f*_b_ increases. This trend can be explained without assuming explicit envelope extraction (Budde et al., 2023). Because the TI waveform contains two frequency components, each component yields a slightly different extracellular potential distribution through σ^*^(*f*) = σ+*j*2π*fε*. As *f*_b_ increases, the separation between *f*_1_ and *f*_2_ increases, which slightly reduces the constructive interference of the resulting fields along the axon and thereby decreases AF_max_. In our parameter range, the difference in |σ^*^| between *f*_1_ and *f*_2_ is on the order of 1% in the skin, while it is negligible in deeper layers (SAT and bone), consistent with the small changes observed in AF_max_.

The axon dynamics simulation revealed phasic APs at the beat frequency, consistent with previous studies (Mirzakhalili et al., 2020; Budde et al., 2023), and the amplitude of threshold current increases exponentially with carrier frequency for human peripheral nerve stimulation via TIES (Opančar et al., 2025). Figure 3b shows that our simulation reproduced these phasic APs at the beat frequency. Figure 10 shows the scaling factors of the amplitude, which increase exponentially with carrier frequency. These scaling factors reflect the sensitivity to stimulation and vary with axon orientation.

For *x*-oriented axons at *f*_c_ = 4 kHz, a distinct behavior was observed: the AP distribution exhibited a steep decline. The scaling factor under this condition is two orders of magnitude larger than that under the other conditions, making activation more difficult. This suppression of AP generation at higher carrier frequencies may be attributed to conduction block. This observation is consistent with the study using the same simulation framework (Mirzakhalili et al., 2020).

Regarding consistency with previous studies, psychophysical experiments reported no tactile perception at *f*_c_ = 1 kHz and *f*_b_ = 0 Hz (Lim et al., 2024). Direct comparison is limited by differences in the electrode configuration.

However, our simulation predicted non-zero AP counts under this condition. This discrepancy may arise from differences in electrode geometry or the threshold criteria used for perceptual detection. Our observation of PF = 0 at *f*_c_≥2 kHz without low-frequency modulation matches previous brain experiment results (Grossman et al., 2017). Moreover, our results suggest that the beat frequency also influences AF_max_ through frequency-dependent tissue admittance. Psychophysical experimental results showing the beat frequency affects the threshold current (Lim et al., 2024) support this interpretation. The scaling factors *S*(*f*_c_, *f*_b_) capture the relative ease of activation across orientations under a fixed current.

The evaluation metrics, PF and PI, agree with the principle that perceptual profiles reflect recruitment-dominant populations and their firing rates. Brain modeling revealed that the amplitude ratio affects PF of axons partially perpendicular to the skin (Gomez-Tames et al., 2021), supporting the parametric adjustment of axon activation via TIES. Psychophysical experiment reported that the peak developed force during TIES of peripheral nerves was maximal at *f*_c_ = 2 kHz and decreased at higher carrier frequencies (4 and 6 kHz) (Osorio et al., 2025). Although direct comparison is limited because the carrier frequencies examined differ between studies, a similar trend was observed in our simulation: PI was higher at *f*_c_ = 2or3 kHz than at *f*_c_ = 1or4 kHz. This agreement supports the validity of our simulation approach for predicting perceptual outcomes in mechanoreceptive fibers.

Despite its simplifications, our simulation framework successfully quantified differences in tactile perception. However, our computational model has some limitations. We used a simplified glabrous skin and axon geometry and an axon cable equation. An anatomically accurate fingertip is rounded and includes additional structures. Here, we approximated the electrode contact as locally flat, and the axon was a straight path. Moreover, Aβ fibers can be represented more precisely by MRG (McIntyre et al., 2002) or mechanoreceptive models (Kaihua et al., 2017). The carrier frequency dependent beat frequency sensitivity reflects complex multilayer dispersion. We expect qualitative trends to generalize, but quantitative validation through psychophysical experiments is necessary.

Compared with TES, TIES has the potential to shape PF and PI without changing the amplitude or repositioning electrodes. In TES, increasing the amplitude broadens activation and may recruit nociceptive C-fibers, causing discomfort, whereas electrode rearrangement compromises compactness.

In contrast, TIES enables parametric adjustment of perceptual profiles through carrier and beat frequency selection.

Furthermore, our study focused on Aβ fibers. Regarding nociceptors (unmyelinated C-fibers and myelinated Aδ fibers), their biophysical parameters differ substantially from those of Aβ fibers. Extending our framework to incorporate nociceptor models would provide further insight into pain perception and the potential for discomfort reduction under TIES, particularly in relation to conduction block.

## 5 Conclusion

This study systematically characterized TIES frequency parameters in mechanoreceptive fibers using FEM simulation and axon dynamics simulations with three orthogonal axon arrays. The carrier frequency sets the bounds on PF and PI, whereas the beat frequency tunes the PF and PI within these bounds. Axon orientation also influences activation thresholds and dynamics, underscoring the need for multiple orientation analysis in peripheral models. These findings provide quantitative guidelines for frequency-based tuning, thus enabling the design of compact electrotactile interfaces by reducing reliance on electrode reconfiguration or amplitude calibration. Although simplified, our model reveals the effects of carrier and beat frequencies on axon activation vary with axon orientation. Our model reveals the roles of multilayer dispersion and orientation-dependent recruitment, thereby providing a foundation for TIES-based electrotactile interfaces. Greater anatomical fidelity, refined axon models, and psychophysical validation will further enable sophisticated haptic rendering in VR/AR.

## Data Availability

The raw data supporting the conclusions of this article will be made available by the authors, without undue reservation.
